# Impact of integrated nursing care on rehabilitation process of inpatients with multiple myeloma in an academic hematology department

**DOI:** 10.1097/MD.0000000000046303

**Published:** 2025-12-19

**Authors:** Yangyang Wan, Xingfang Wu, Yangli Ou

**Affiliations:** a Hematology Department, West China Hospital of Sichuan University, Chengdu City, Sichuan Province, China; b Hematology Department, Guihang Guiyang Hospital, Guiyang City, Guizhou Province, China; c Department of Nursing, The First Affiliated Hospital of Chongqing Medical Universit, Yuzhong District, Chongqing, China.

**Keywords:** complication, comprehensive nursing mode, myeloma, satisfaction

## Abstract

Multiple myeloma (MM) is a complex hematologic malignancy often associated with complications and challenges in recovery. The research on the specific effects and influencing factors associated with the application of comprehensive nursing mode in hospitalized patients with multiple myeloma in hematology department is still limited. To explore the positive for potential benefits of a comprehensive nursing model. To collect the clinical data of patients with multiple myeloma who received routine chemotherapy in hematology department of our hospital from March 2020 to June 2024. A total of 136 patients were enrolled, including 77 males and 59 females. The patients were randomly divided into the experimental group and the control group, and received either a comprehensive or routine nursing model. Data on hospitalization duration, complication rates, and health-related quality of life during the diagnostic and treatment process were collected for both groups, followed by an analysis of the scientific validity of the comprehensive nursing model. The analysis shows that the hospital stay of patients in the experimental group (4.90 ± 0.31days) is shorter than that in the control group (5.06 ± 0.24 days), and the difference is statistically significant (*P* <.05). The quality of life score and the compliance of patients with treatment and nursing activities during hospitalization in the experimental group were better than those in the control group, and the difference was statistically significant (*P* <.05). The incidence of complications in the experimental group was significantly lower than that in the control group, and the satisfaction of patients in the experimental group with the diagnosis and treatment process was also better than that in the control group. Comprehensive nursing mode is a more effective nursing mode for inpatients, which can effectively reduce the hospitalization time of patients and the incidence of complications, and can improve patients’ satisfaction with nursing staff. In practice, we need to constantly explore and improve this method to adapt to different application scenarios and needs.

## 1. Introduction

Multiple myeloma (MM) is a complicated and fatal hematologic malignancy, which usually manifests as abnormal proliferation of malignant plasma cells in bone marrow, characterized by clinical symptoms such as bone pain, anemia, hypercalcemia and renal function damage.^[1–5]^ MM is predominantly diagnosed among individuals aged between 65 and 75,^[6]^ with the median age being 70.^[7]^ In 2019, the global count of newly documented cases of MM stood at 155,688, reflecting an age-standardized incidence rate of 1.92 per 100,000 individuals.^[8]^ This statistic denotes a roughly 1.36-fold escalation from the age-standardized incidence rate of 1.72 per 100,000 recorded in 1990.^[8]^ In 2022, the International Agency for Research on Cancer disclosed that the 5-year incidence rates stood at 1.5 per 100,000 in Africa, 4.3 per 100,000 in Asia, 20 per 100,000 in Europe, and a notable 31.3 per 100,000 in North America.^[9]^ Authoritative studies further reveal that the incidence of MM peaks in North America, Australia, New Zealand, and Europe, in stark contrast to its significantly lower rates in Asia (excluding West Asia).^[10]^ In 2020, it was estimated that MM resulted in 117,077 fatalities globally, translating to a mortality rate of 1.14 per 100,000 individuals.^[11]^ MM persists as an incurable malignant neoplasm of the hematopoietic system, with most patients eventually suffering from relapse and acquiring resistance to existing treatments. Present therapeutic strategies for MM include the utilization of immunomodulatory agents, monoclonal antibodies, T-cell redirection immunotherapy, and hematopoietic stem cell transplantation.^[9]^

Although treatment methods (such as targeted therapy and immunotherapy) have made progress in recent years, patients are faced with significant rehabilitation challenges during hospitalization due to the chronic progress of the disease and treatment-related side effects, such as drug side effects, infection risks and functional impairments, including bone - related movement dysfunction, hematologic dysfunction (e.g., anemia and thrombocytopenia).^[1,2]^ Traditional nursing models often focus on single-level medical interventions,^[12,13]^ making it difficult to fully address the complex needs of patients with MM. Therefore, the introduction of comprehensive nursing mode has brought new hope to this field. The comprehensive nursing model emphasizes interdisciplinary cooperation, including medical care, nursing, psychological and social support, aiming at improving the overall rehabilitation effect of patients through all-round intervention.^[14]^ Previous studies have shown that this model can effectively improve the quality of life of patients, reduce hospitalization time and reduce the incidence of complications.^[15–17]^ At present, the research on the influence of comprehensive nursing mode on patients with MM at home and abroad mainly focuses on its effect on patients’ quality of life, pain management and mental health. For example, Choon-Quinones and others found that the comprehensive nursing model can significantly improve the quality of life of patients with MM and reduce the adverse reactions related to treatment.^[18]^ However, in China, there are few systematic studies in this field, and there is still a lack of specific data and practical guidance for patients in China. The purpose of this study is to fill the gap in the existing literature by analyzing the application effect and influencing factors of comprehensive nursing mode in hospitalized patients with MM in hematology department. By systematically evaluating the influence of comprehensive nursing mode on patients’ rehabilitation, this study hopes to provide scientific basis for improving nursing practice and formulating more individualized treatment plans.

## 2. Materials and methods

### 2.1. Research objects

In this study, a prospective study was used to collect the clinical data of patients with MM who received conventional chemotherapy in the hematology department of our hospital from March 2020 to June 2024. A total of 136 patients were enrolled, including 77 males and 59 females. This study was approved by the Ethics Committee of our hospital.

### 2.2. Research methods

136 patients collected in this study were randomly divided into 2 groups. Among them, 68 cases in the control group adopted conventional nursing mode, while 68 cases in the experimental group adopted comprehensive nursing mode. The related factors of hospitalization time, complications, quality of life and satisfaction with medical services between the 2 groups were collected and statistically analyzed.

### 2.3. Collect indicators

This study collected patients’ gender, age (years), BMI, hospitalization time (days), complications, quality of life and satisfaction with medical services.

### 2.4. Groups

#### 2.4.1. Control group

The Control group adopt the conventional nursing mode, which encompasses patiently introducing knowledge about MM to patients, closely monitoring their vital signs, guiding them to follow the doctor’s advice regarding diet and medication, and providing routine psychological and nutritional support. Also, standardize aseptic operation and dressing change in nursing process to reduce the risk of infection and drug extravasation caused by improper operation.

#### 2.4.2. Experimental group

##### 2.4.2.1. Comprehensive evaluation

Comprehensive evaluation of patients with MM after admission to understand the patients’ cognition of the disease; Then, according to the evaluation results, the nursing plan was formulated and provide personalized nutritional support programmes.

##### 2.4.2.2. Health education

Explain to patients the daily nursing care of MM, related treatment precautions and necessary emergency treatment methods.

##### 2.4.2.3. Psychological nursing

Provide systematic psychological support. After admission, some patients often have negative emotions such as nervousness and anxiety because they are too worried about the progress of the disease. Explain to the patient the necessity of treatment, emphasizing that adhering to medical advice and cooperating with standard care can significantly reduce the risk of adverse events while maximizing their ability to maintain normal work and daily life. Concurrently, inform them of potential risks and corresponding measures to alleviate anxiety.

##### 2.4.2.4. Improve nursing quality

Improve the professional skills and communication skills of nursing staff through training, case discussion and skill competition. Standardize aseptic operation and dressing change in nursing process to reduce the risk of infection and drug extravasation caused by improper operation. Reduce the occurrence of events such as uncooperative and dissatisfied patients caused by poor communication.

### 2.5. The relevant issues mentioned in this study

#### 2.5.1. Nutritional risk assessment

In this study, the nutrition risk screening 2002 (NRS2002) scale was utilized to evaluate patients’ nutritional status.^[19]^ The scale yields a total score ranging from 0 to 7 points, where a score of ≥3 points signifies nutritional risk. For additional information, kindly consult Supplementary Material 1, Supplemental Digital Content, https://links.lww.com/MD/Q807.

For patients at nutritional risk, personalized nutritional support should be provided according to the patient’s specific circumstances. Personalised nutritional guidance is a practice model that develops tailored nutritional intervention plans based on an individual’s health status, metabolic characteristics, dietary preferences, and lifestyle. Its core lies in meeting the specific nutritional requirements of diverse populations – such as those with chronic conditions, athletes, and the elderly – through precise assessments (including body composition analysis, nutrient deficiency testing, and metabolic capacity evaluation) and dynamic adjustments, rather than employing standardized dietary advice that applies uniformly to all. In clinical nursing, personalized nutritional guidance is led by clinical nutritionists in collaboration with other professionals (such as physicians, psychologists, and exercise physiotherapists), forming a closed-loop system of “assessment-intervention-monitoring-adjustment.”

#### 2.5.2. Psychological assessment

In this study, the self-rating depression scale (SDS) was utilized to assess the severity of depressive symptoms in patients,^[20]^ while the self-rating anxiety scale (SAS) was employed to evaluate their anxiety levels.^[21]^ See Supplementary Material 2, Supplemental Digital Content, https://links.lww.com/MD/Q807 for details.

For patients experiencing anxiety or depression, systematic psychological support should be provided. Systemic psychological support constitutes a comprehensive mental health service model centered on patients’ psychological needs. Through structured assessment, multi-stage interventions, and interdisciplinary collaboration (integrating medical, psychological, and social resources), it establishes a closed-loop system encompassing “assessment – tiered interventions – multi-professional collaboration – long-term follow-up.” This approach aims to deliver personalized, end-to-end, sustainable psychological support, thereby enhancing patients’ quality of life and rehabilitation outcomes. Concurrently, it optimizes the allocation of healthcare resources and promotes equitable social health outcomes.

#### 2.5.3. Pain assessment

This study employed the visual analogue scale to assess patients’ pain status.^[22]^ A 10 cm straight line is employed, with 1 end denoting the absence of pain (0 points) and the other end signifying extreme pain (10 points). The patient indicates the relevant position on the line corresponding to their perceived pain level, and the clinician evaluates the pain severity based on the distance between the marked point and the no-pain end.

#### 2.5.4. Evaluation of patients’ satisfaction with nursing staff

This study employed the Newcastle Satisfaction with Nursing Scale to assess patients’ satisfaction with nursing staff.^[23]^ This scale utilizes a 5-point Likert scale, wherein patients choose the appropriate level according to their individual experiences. A 5-point scale spans from 1 to 5, with each point corresponding to: Completely dissatisfied, dissatisfied, neutral, satisfied, and completely satisfied, respectively. A cumulative score of <60 signifies patient dissatisfaction with the care quality, whereas a score ranging from 60 to 95 denotes patient satisfaction with the service quality rendered by the nursing staff. See Supplementary Material 3, Supplemental Digital Content, https://links.lww.com/MD/Q807 for details.

#### 2.5.5. Quality of life assessment

In this study, the quality of life was evaluated with reference to the Comprehensive Quality of Life Assessment Scale (GQOL-74). The GQOL-74 is a standardized tool specifically designed to assess the quality of life among patients with chronic conditions and special populations. Its core value lies in providing scientific evidence for clinical interventions and health management through multidimensional quantitative analysis. The following sections outline its structure, functionality, application scenarios, and advantages.

#### 2.5.6. Standardisation of the integrated care model

To guarantee that patients in the experimental group received standardized and comprehensive care, this study devised a meticulously crafted checklist tool.^[24]^ This tool specifically incorporated elements such as disease education topics discussed with patients, pain assessment instruments and corresponding pain management strategies, nutritional questionnaires accompanied by pertinent guidance, as well as an evaluation of patients’ social support networks. See Supplementary Material 4, Supplemental Digital Content, https://links.lww.com/MD/Q807 for details. The GQOL-74 comprises 74 items, categorized into 4 dimensions: physical functioning, psychological functioning, social functioning, and material living conditions.

### 2.6. Statistical method

The data were statistically analyzed using SPSS 25.0 statistical software (Chicago). Measurement data is represented by average number ± standard deviation; Categorical variables are expressed as counts (percentages). When the measurement data obey the normal distribution, the paired t test is used for analysis. When the measurement data does not obey the normal distribution, it is represented by median (M) and interquartile interval (Q), and the rank sum test is used. Descriptive statistical analysis and one-way ANOVA were used to analyze the related indexes of comprehensive nursing mode. Chi-square test was used to verify the value of comprehensive nursing mode in improving quality of life and satisfaction with medical services. *P* <.05 is statistically significant.

## 3. Results

### 3.1. Comparison of baseline characteristics

Patients in both groups were randomly divided into 2 groups: 40 males and 28 females in the experimental group; There were 37 males and 31 females in the control group, *P* = .604 >0.05, and there was no significant difference between the 2 groups. Comparing the age and BMI of the 2 groups, the *P*-values were all >.05, and there was no statistical difference between the 2 groups (*P* >.05). The 2 groups are balanced and comparable at the baseline data level. See Table 1.

**Table 1:** Comparison of baseline characteristics.

Variables	Total (n = 136)	Control group (n = 68)	Experimental group (n = 68)	Statistic	*P*
Age, mean ± SD	51.32 ± 8.64	51.78 ± 8.69	50.87 ± 8.63	*t* = 0.61	.540
BMI, mean ± SD	26.05 ± 1.01	26.06 ± 0.96	26.03 ± 1.07	*t* = 0.17	.865
Gender, n (%)					
1	77 (56.62)	37 (54.41)	40 (58.82)	χ^2^ = 0.27	.604
0	59 (43.38)	31 (45.59)	28 (41.18)		

### 3.2. Comparison of hospitalization time, quality of life and compliance between the 2 groups

In this study, the quality of life was evaluated with reference to the Comprehensive Quality of Life Assessment Scale (GQOL-74).^[24]^ Compared with the control group (5.06 ± 0.24 days), the hospitalization time of patients in the experimental group (4.90 ± 0.31 days) was shorter, and the difference was statistically significant (*P* <.05). The quality of life score and the compliance of patients with treatment and nursing activities during hospitalization in the experimental group were better than those in the control group, and the difference was statistically significant (*P* <.05). See Table 2.

**Table 2:** Comparison of patient hospitalization information.

Variables	Total (n = 136)	Experimental group (n = 68)	Control group (n = 68)	Statistic	*P*
Hospitalization time (d)	4.98 ± 0.28	4.90 ± 0.31	5.06 ± 0.24	*t* = −3.45	<.001
Quality of life	76.65 ± 10.61	78.87 ± 7.19	74.43 ± 12.84	*t* = 2.49	.014
Compliance, n (%)					
0	123 (90.44)	66 (97.06)	57 (83.82)	χ^2^ = 6.89	.009
1	13 (9.56)	2 (2.94)	11 (16.18)	–	–
Degree of satisfaction, n (%)					
0	126 (92.65)	66 (97.06)	60 (88.24)	*χ*^2^ = 3.89	.049
1	10 (7.35)	2 (2.94)	8 (11.76)		

### 3.3. Incidence of complications

By consulting the patient’s medical records, we can get the complications during hospitalization. Among them, there were 4 cases of thrombocytopenia, 7 cases of diarrhea, 5 cases of constipation and 8 cases of nausea and vomiting in the control group, totaling 24 cases. In the experimental group, there were 2 cases of thrombocytopenia, 2 cases of diarrhea, 1 case of constipation and 2 cases of nausea and vomiting, totaling 7 cases. Comparing all the complications between the 2 groups, the incidence of complications in the experimental group was significantly lower than that in the control group, and the difference was statistically significant (*P* <.05). See Table 3.

**Table 3:** Incidence of complications.

	Thrombocytopenia	Diarrhea	Constipation	Nausea and vomiting	Total
Control group	4 (5.88%)	7 (10.29%)	5 (7.35%)	8 (11.76%)	24 (35.29%)
Experimental group	2 (2.94%)	2 (2.94%)	1 (1.47%)	2 (2.94%)	7 (10.29%)
Statistic	–	–	–	–	χ^2^ = 12.07
*P*	–	–	–	–	**<.001**

### 3.4. Patient satisfaction survey

Before leaving the hospital, we investigated the patients and asked them about their satisfaction with the hospital’s diagnosis and treatment activities. It was found that the satisfaction of patients in the experimental group was significantly higher than that in the control group, and the difference was statistically significant (*P* <.05). See Table 2.

## 4. Discussion

MM is a complicated hematological malignant tumor, and its treatment process is often accompanied by many symptoms and complications, and the rehabilitation process is relatively long. Traditional nursing mode often pays attention to single treatment and nursing, while comprehensive nursing mode adopts multi-angle nursing intervention and support. It is expected to play a greater role in improving patients’ quality of life, shortening rehabilitation time and improving treatment effect. In this study, we discussed the practical impact of the comprehensive nursing model in the rehabilitation of patients with MM, and analyzed the key factors affecting its effectiveness. Comprehensive nursing mode includes not only routine clinical nursing, but also psychological support, nutritional intervention, symptom management and social support. We hope that through the in-depth analysis of these factors, we can reveal the specific influence of comprehensive nursing mode on the rehabilitation of patients with MM.

### 4.1. Effectiveness of comprehensive nursing model

Our research shows that the comprehensive nursing mode has a significantly positive impact on the rehabilitation of patients with MM. This is consistent with the findings in the existing literature. For example, recent research shows that the comprehensive nursing model can effectively improve the quality of life and compliance of patients through multidisciplinary teamwork, personalized nursing plan and patient education.^[25–28]^ Our study further verified the effectiveness of these models in patients with MM, and showed specific improvements in hospitalization time, quality of life and compliance.

At present, the research shows that systematic psychological intervention has significantly improved the mental health of patients, thus enhancing the overall treatment effect. This shows that psychological support not only helps to relieve patients’ anxiety and depression, but also enhances the compliance of treatment and the enthusiasm of patients.^[29,30]^ In addition, regular nursing evaluation is the core part of comprehensive nursing model,^[31]^ it can discover patients’ health problems and treatment responses in time, allowing the nursing team to promptly adjust care plans and intervention measures. This dynamic adjustment is helpful to optimize the rehabilitation path of patients, reduce the occurrence of complications and improve the overall treatment effect. At the same time, nutrition guidance is also an important intervention measure in comprehensive nursing mode. It is found that individualized nutritional intervention can improve the nutritional status and immune function of patients and reduce the side effects of treatment, such as weight loss and infection risk.^[32]^ By providing personalized dietary advice and supplementing appropriate nutrients, patients can maintain their physical strength and improve their overall health.

To sum up, the comprehensive nursing model has effectively promoted the rehabilitation of patients with MM through multi-level and multi-faceted intervention measures. Systematic psychological support, regular nursing evaluation and personalized nutrition guidance have significantly improved the quality of life and therapeutic effect of patients, which has provided strong support for clinical practice.

### 4.2. Shortening hospitalization time, improving quality of life, reducing complications and improving patient satisfaction

Our study confirmed that the hospitalization time of the experimental group was significantly shortened, which may be related to improving the quality of nursing, optimizing the treatment plan and timely intervention in the comprehensive nursing model. Considering that high-quality nursing patients can actively cooperate with treatment, they can shorten the hospitalization time by reducing the occurrence of complications and improving patients’ ability to self-manage. Our research results confirm this view, and our research can provide intervention strategies for concrete practice. However, future research needs to further explore whether the shortening of hospitalization time is related to other factors, such as the complexity of the patient’s condition and the choice of treatment plan.

In addition, this study found that the comprehensive nursing model significantly improved the patients’ quality of life score. The latest research shows that comprehensive nursing mode can effectively improve the quality of life by improving patients’ psychological state, improving the accessibility of support system and promoting the recovery of physical function.^[33]^ Analyzing its internal mechanism, we may consider the following reasons: Comprehensive nursing mode can improve patients’ physical and mental state from multiple dimensions, understand patients’ health status and needs, and adjust nursing plans according to evaluation results. This individualized nursing method helps to better meet the specific needs of patients, thus improving their quality of life. In addition, this model usually includes various interventions such as managing pain, controlling symptoms and alleviating side effects. All these are helpful to relieve patients’ pain, improve their quality of life and improve their satisfaction.

Finally, the results show that the incidence of complications in the experimental group is significantly lower than that in the control group. The comprehensive nursing mode effectively reduces the incidence of complications of patients through regular monitoring, early intervention, personalized nursing plan, multidisciplinary teamwork, health education and self-management, holistic nursing and comprehensive management. The author considers that the reduction of complications is related to the specific nursing strategies in the comprehensive nursing mode, such as early identification, individualized nursing and continuous monitoring. The comprehensive application of these measures optimizes the nursing process, improves the overall health level of patients, and then significantly improves the treatment effect and quality of life. For instance, the integrated care model can reduce the incidence of severe thrombocytopenia by implementing preventive measures – such as employing thrombopoietic agents and minimizing invasive procedures – following advance assessment of patient risks, including pre-chemotherapy platelet counts and prior bleeding history. For patients who have developed thrombocytopenia, prompt monitoring for bleeding symptoms, adjusting activity levels (such as bed rest), and avoiding anticoagulant medications can reduce the risk of bleeding. These nursing interventions may consequently influence the clinical grading of thrombocytopenia. Concurrently, the integrated care model can further alleviate patients’ anxiety by providing systematic psychological support. This indirectly improves treatment adherence, thereby enhancing therapeutic outcomes and reducing the incidence of complications.

### 4.3. Limitations

The sample size of this study is relatively small, which may limit the wide applicability of the research results. Although we adopted the method of random distribution in both the experimental group and the control group, the insufficient sample size may lead to the instability of statistical results. This study is constrained by its single-center, short-term observational design and the absence of post-discharge follow-up data. Consequently, it presents limitations in analyzing the impact of treatment on quality of life, functional recovery, and long-term healthcare resource utilization (such as readmissions). Future research should extend follow-up periods to more comprehensively evaluate the long-term benefits of intervention measures. This study exhibits gaps in key characteristics regarding patients’ disease status and comorbidities at the time of diagnosis. Consequently, when analyzing the efficacy of rehabilitation nursing, it is impossible to comprehensively account for the underlying disease and comorbidities’ influence. This leads to assessment bias, compromising the judgement regarding optimization of rehabilitation nursing strategies and undermining the reliability of research conclusions. Although this design can control some confounding factors, the research results of single center may be influenced by the specific institutional environment and the characteristics of nursing team. Therefore, the universality of the research results may be limited. At the same time, the observation period of this study is short, and it may not be possible to comprehensively evaluate the influence of comprehensive nursing mode on patients’ long-term rehabilitation and quality of life. This study did not stratify patients according to disease staging, chemotherapy type, or treatment timing (e.g., whether all patients were enrolled within the first chemotherapy cycle). Consequently, potential selection bias exists in this research. In order to further verify the effect of comprehensive nursing mode, it is suggested that future research should include more samples and multi-center design, extend the observation period, and evaluate with biomarkers and objective indicators. In addition, a detailed stratified analysis of patients in different subgroups to explore the influence of individual differences on nursing effect will also provide valuable basis for the optimization of comprehensive nursing model; The data should also be followed up over time; long-term follow-up studies can provide a more comprehensive assessment of the long-term effects of the care model, and future studies should consider extending the observation period to obtain long-term data.

## 5. Conclusions

Comprehensive nursing mode is a more effective nursing mode for inpatients, which can improve the overall health level and rehabilitation process of patients through multidisciplinary cooperation and personalized intervention measures. Comprehensive nursing mode can effectively reduce the hospitalization time of patients, reduce the incidence of complications, and improve the satisfaction of patients with nursing staff. Therefore, implementing the comprehensive nursing mode in clinical settings will contribute to enhancing the nursing outcomes for patients with MM. Simultaneously, it is essential to continually investigate and enhance this approach to cater to the varying application scenarios, which may include different hospital environments, care team compositions, and patient - specific factors such as age, comorbidities, and personal preferences, as well as the distinct needs arising from these individual characteristics. However, the sustainability and stability of the nursing outcomes require further investigation. In light of this, we earnestly encourage subsequent research to broaden the scope of evaluation, focusing on examining the multidimensional impacts of integrated care over extended periods – such as throughout the patient’s entire treatment journey. This should encompass dynamic shifts in physiological indicators, long-term adjustments in psychological states, and sustained improvements in quality of life. Such an approach will provide a more robust and comprehensive scientific basis for optimizing and promoting integrated care models.

## Acknowledgments

Thank you for the support of all the medical staff in the Hematology Department of West China Hospital of Sichuan University.

## Author contributions

**Data curation:** Xingfang Wu.

**Writing – original draft:** Yangyang Wan.

**Writing – review & editing:** Yangyang Wan, Yangli Ou.

## Supplementary Material


