# Supplementary material for: Impact of integrated nursing care on rehabilitation process of inpatients with multiple myeloma in an academic hematology department
Source: Medicine (Baltimore). 2025 Dec 19;104(51):e46303. doi: 10.1097/MD.0000000000046303 (PMC12727334; doi:10.1097/MD.0000000000046303)
Supplement: Supplementary file 1 [file medi-104-e46303-s001.pdf]

Nutrition Risk Screening 2002 (NRS2002) scale

Category	Marking Scheme (points) and Content	Score
Disease Severity Score	☐ 0 point: No relevant diseases.<br>☐ 1 point: Patients with chronic diseases hospitalized due to complications, who are frail but not bedridden. Protein requirements are slightly increased but can be met with oral supplements.<br>☐ 2 points: Patients requiring bed rest (e.g., after major abdominal surgery). Protein requirements are increased but can still be partially met through enteral/parenteral nutrition.<br>☐ 3 points: Patients in intensive care units on mechanical ventilation. Protein requirements are significantly increased and cannot be fully met through enteral/parenteral nutrition, though these interventions can reduce protein catabolism and nitrogen loss.	

Nutritional Status Impairment Score	☐ 0 point: Normal nutritional status.<br>☐ 1 point: Weight loss >5% within the past 3 months, or Food intake 50%-75% of normal requirements in the past week.<br>☐ 2 points: Weight loss >5% within the past 2 months, or Food intake 25%-50% of normal requirements in the past week.<br>☐ 3 points: Weight loss >5% within the past 1 month and total weight loss >15% within the past 3 months, or BMI <18.5, or Food intake 0%-25% of normal requirements in the past week.	
Age assessment	☐ 0 point: Age <70 years.<br>☐ 1 point: Age ≥70 years.	
Total Score		
Screening recommendations	Total score < 3 points; reassessment in one week's time.<br>3 points ≤ Total score < 5 points: Provide nutritional education and, if necessary, consult the Nutrition	

	Department.<br>Total score ≥ 5 points: Conduct nutritional education; a consultation with the Nutrition Department is required.

The Self-Rating Depression Scale(SDS)

Items / degree	A Little of the Time	Some of the Time	Good Part of the TIme	Most of the TIme
1.I teel down-hearted and blue				
2.Morning is when I feel the best				
3.Ihave crying spells or feel like ft				
4.Ihave trouble sleeping at night				
5.I eat as much as I used tu				
6.I still enjoy sex				
7.Inotice that I am losing welght				
8.Ihave trouble with constipation				
9.My heart beats faster than usual				
10.I get tired for no reason				
11.My mind is as clear as it used to be				
12.I find it easy to do the things I used to				
13.I am restless and can't keep stili				
14.I teel hopeful about the future				
15.Iam more Irritable than usual				
16.I fAnd It easy to make decis1ons				
17.I feel that I am useful and				

needed

18.Mylife is pretty full

19.I feel that others would be better ofifI were dead

20.I still enjoy the things I used to do

Note: The SDS scale comprises 20 items, covering dimensions such as low mood, physical discomfort, and self-perception. A 4-point rating scale (1–4) is employed, with each item corresponding to four options:

1 point: No or very little time

2 points: A small portion of the time

3 points: A considerable amount of time

4 points: Most or all of the time

Questions 2, 5, 6, 11, 12, 14, 16, 17, 18 and 20 are reverse-scoring questions. The marks must be inverted: a mark of 1 counts as 4 points, a mark of 2 counts as 3 points, and so on. The inverted scores are then added to the marks from the other questions.

Add the scores for all 20 questions to obtain the raw score (range 20–80 points).

Standardised score = Raw score × 1.25 (range 25–100 points), used to standardise comparisons across different versions.

The cut-off score is 53 points (some studies adjust the threshold to 50 points, requiring assessment based on specific contexts).

≤52 points: No depression or mild symptoms.

53–62 points: Mild depression, potentially involving mood swings or sleep disturbances.

63–72 points: Moderate depression, accompanied by significant physical or psychological distress.

≥73 points: Severe depression, requiring prompt medical intervention.

The Self-rating Anxiety Scale(SAS)

Items \ degree	None OR A little of the time	Some of the time	Good part of the time	Most OR All of the time
1.I feel more nervous and anxious than usual				
2.I feel afraid for no reason at all				
3.I get upset easily or feel panicky				
4.I feel like I'm falling apart and going to pieces				
5.I feel that everything is all right and nothing bad will happen				
6.My arms and legs shake and tremble				
7.I am bothered by headaches, neck and back pains				
8.I feel weak and get tired easily				
9.I feel calm and can sit still easily				
10.I can feel my heart beating fast				
11.I am bothered by dizzy spells				
12.I have fainting spells or feel like it				
13.I can breathe in and out easily				
14.I get feelings of numbness and tingling in my fingers,toes				
15.I am bothered by stomachaches or indigestion				

16.I have to empty my bladder often

17.My hands are usually dry and warm

18.My face gets hot and blushes

19.I fall asleep easily and get a good night's rest

20.I have nightmares

Note: The SAS scale comprises 20 items covering core symptoms of anxiety (such as tension, uneasiness, fear, insomnia, and physical discomfort), and is used to assess an individual's anxiety levels over the preceding week. Each item is rated on a four-point scale (1–4) according to the frequency of symptom occurrence, with the following options:

1 point: No or very little time

2 points: A small portion of the time

3 points: A considerable amount of time

4 points: Most or all of the time

Questions 5, 9, 13, 17 and 19 are reverse-scoring questions. The marks must be inverted (i.e. 1 mark counts as 4 points, 2 marks as 3 points, 3 marks as 2 points, and 4 marks as 1 point) before being added to the scores from other questions.

Raw score: Sum the marks for all 20 questions to obtain the raw score (range 20–80 points).

Standardised score: Multiply the raw score by 1.25 and round to the nearest whole number (range 25–100 points), used to standardise comparisons across different versions.

Formula: Standardised score = Raw score × 1.25

Example: Raw score 60 → Standardised score = 60 × 1.25 = 75 points

≤49 indicates no anxiety or mild symptoms; 50–59 indicates mild anxiety; 60–69 indicates moderate anxiety; ≥70 indicates severe anxiety.

Newcastle satisfaction with nursing scale

Serial number	Question	Very dissatisfied	Dissatisfied	Generally	Satisfy	Very satisfied
1	Time spent by nurses	1	2	3	4	5
2	Nurse’s ability to work	1	2	3	4	5
3	There is always a nurse by your side when you need it	1	2	3	4	5
4	How well the nurse knows about your care	1	2	3	4	5
5	When you call a nurse, how fast they arrive	1	2	3	4	5
6	The way the nurse treats you makes you feel at home	1	2	3	4	5
7	The amount of information the nurse can give you about illness and treatment	1	2	3	4	5
8	Number of ward visits by nurses	1	2	3	4	5
9	Help provided by nurses	1	2	3	4	5
10	How the nurse explains the problem to you	1	2	3	4	5
11	The extent to which the nurse reassures your relative or friend	1	2	3	4	5
12	Nurses' attitudes towards their own work	1	2	3	4	5
13	The type of information the nurse gives you about illness and treatment	1	2	3	4	5
14	The level of respect the nurse treats you during the nursing process	1	2	3	4	5
15	How nurses listen to your concerns and concerns	1	2	3	4	5

16	The degree of freedom the nurse gives you during your hospital stay, subject to the rules and regulations	1	2	3	4	5
17	How willing the nurse is to respond to your request	1	2	3	4	5
18	How well the nurse protects your privacy	1	2	3	4	5
19	Nurses understand your needs	1	2	3	4	5

Tools employed by the comprehensive nursing model (Experimental group) group

1. Disease-Related Education Programme Tools

1.1 Foundational Knowledge Module on Disease

1.1.1 Core Content

Disease Definition: Explain in layman's terms that ‘multiple myeloma is a malignant tumour of the blood system caused by abnormal proliferation of plasma cells.’

Pathogenesis: Illustrate using metaphors (e.g., ‘malignant cells overgrow within the bone marrow, crowding out normal blood cells’).

Typical Symptoms: Illustrated comparison chart showing bone pain (red-highlighted painful areas), anaemia (pallor diagram), and renal dysfunction (case study of increased urinary foam).

1.1.2 Visualisation tools

Symptom self-assessment card: Double-sided design. Front side lists symptoms (bone pain/fatigue/recurrent infections). Back side indicates urgent medical attention criteria (e.g. persistent high fever >38.5°C, excruciating pain preventing walking).

Treatment Protocol:

Diagnosis (bone marrow biopsy) → Induction chemotherapy (4–6 cycles) → Assessment (MRD testing) → Maintenance therapy (oral medication)

└─ Relapse management (new chemotherapy regimen/CAR-T therapy)

1.2 Treatment Management Module

1.2.1 Chemotherapy Cycle Management Schedule

Horizontal axis: Marking time points for cycles 1–6

Vertical columns:

Medication records (drug name, dosage, administration method)

Side effect alerts (e.g., peripheral neuropathy potentially occurring after bortezomib cycles)

Follow-up tests (complete blood count, β2-microglobulin testing schedule)

1.2.2 Medication Adherence Tool

Pill Organiser Guide:

Divide medications into a 7-compartment pill organiser by chemotherapy cycle (marked daily/weekly)

Special medication notes (e.g. dexamethasone to be taken on an empty stomach upon waking)

Missed Dose Management Flowchart:

Missed dose < 4 hours → Take immediately

Missed dose ≥ 4 hours → Omit this dose; take next dose as scheduled

(Note: Missing doses of immunomodulators may compromise efficacy)

1.3 Complications Prevention Module

1.3.1 Infection Control Manual

Environmental Management:

Household Disinfection Protocol (Chlorine-based disinfectant solution ratio: 500mg/L aqueous solution for wiping surfaces)

Face Mask Wearing Guidelines (Illustrated instructions for appropriate use of N95 masks versus standard medical masks)

Personal Protection:

Illustrated Six-Step Handwashing Method (Including diagrams highlighting fingertips and finger creases for thorough cleansing)

Social Distancing Guidelines to Avoid Crowds (Illustrated examples demonstrating ≥1 metre safe distance)

1.3.2 Bone Event Prevention Tool

Fall Risk Assessment Form

Risk Factors	Rating (0-3 points)
Recent use of dexamethasone	2 points
Bone pain VAS score ≥ 4 points	3 points
Lack of assistive devices	1 point

Intervention measures: Individuals scoring ≥3 points on the total scale shall be provided with a walking frame and scheduled for daily bedside rehabilitation exercises.

1.4 Emergency Response Module

1.4.1 Fever Management Guide

Emergency Contact Card: Front side displays 24-hour emergency hotline and attending physician's contact details. Reverse side outlines fever management protocol:

Body temperature ≥38°C → Immediately administer paracetamol orally (refer to dosage chart)

→ If fever persists after 1 hour → Contact the nurses' station

→ If accompanied by chills/rash → Seek immediate medical attention

1.4.2 Bleeding Management Kit

Contains sterile gauze, elastic bandages, haemostasis instructions (illustrated guide demonstrating 10-minute nasal wing compression for epistaxis)

Emergency mnemonic: ‘Lie flat, elevate, apply pressure, call for help’

1.5 Implementation Method

Initial Education: Within 24 hours of admission, the responsible nurse conducts a one-to-one explanation using the handbook. (Duration: approximately 30 minutes)

Reinforcement Education: Patient education sessions held every Wednesday afternoon, featuring 3D animated demonstrations of disease progression (Duration: 15 minutes)

Effectiveness Assessment: Pre-discharge evaluation using a disease knowledge questionnaire (10 questions covering symptom recognition, medication dosages, etc.). Patients achieving ≥80% accuracy receive a ‘Knowledge Proficiency Certificate’.

2. Pain Assessment and Management Tool

2.1 Pain Assessment

Standardised use of the Visual Analogue Scale (VAS), with provision of graduated sliding rulers (0 – 10 cm).

2.2 Pain Management Protocol

Mild Pain (VAS 1 – 3): Non-steroidal anti-inflammatory drugs (NSAIDs) + Physical relaxation techniques (e.g., guided deep breathing cards)

Moderate Pain (VAS 4 – 6): Weak opioid medication + Guidelines for hot/cold compress application

Severe Pain (VAS ≥7): Strong opioid medication + Pain diary (recording timing, intensity, and alleviating factors)

3. Nutrition Assessment and Guidance Tool

3.1 Nutritional Screening

Employing the NRS 2002 scale (Appendix 1), comprising:

Nutritional Status Impairment Score (0–3 points)

Disease Severity Score (0–3 points)

Age-Adjusted Score (+1 point for ≥60 years)

3.2 Personalised Guidance

Provided based on screening results:

High-risk patients: Nutrition department consultation referral form + bespoke dietary plan (including 3-day sample menu)

Medium-risk patients: Oral nutritional supplement (ONS) usage instructions (brand, dosage, timing)

Low-risk patients: Healthy eating information leaflet (highlighting calcium and protein intake recommendations)

4. Assessment of the Patient's Psychological Condition

In this study, the Self-Rating Depression Scale (SDS) was utilized to assess the severity of depressive symptoms in patients, while the Self-Rating Anxiety Scale (SAS) was employed to evaluate their anxiety levels. See Supplementary Material 2 for details.
